# Long-term delayed emergence after remimazolam-based general anesthesia: a case report

**DOI:** 10.1186/s40981-022-00576-7

**Published:** 2022-10-19

**Authors:** Tsubasa Takemori, Yoshimasa Oyama, Takenori Makino, Seigo Hidaka, Takaaki Kitano

**Affiliations:** Department of Anesthesiology and Intensive Care Medicine, Faculty of Medicine, Oita University, 1-1 Idaigaoka-Hasamamachi, Yufu City, Oita, 879-5593 Japan

**Keywords:** Remimazolam, Flumazenil, Benzodiazepines, Prostatic neoplasms, Prostatectomy

## Abstract

**Background:**

Remimazolam is an ultra-short-acting benzodiazepine anesthetic that is antagonized by flumazenil, and it is typically expected to be applied in anesthesia with the purpose of ensuring early postoperative recovery. We report a case of long-term delayed emergence with re-sedation even after three times of flumazenil administration.

**Case presentation:**

A 71-year-old man was scheduled for a robotic-assisted laparoscopic radical prostatectomy for prostate cancer. We used remimazolam for anesthetic induction and maintenance. The intraoperative bispectral index (BIS) was 30–50. Flumazenil was administered as patient emergence was delayed after surgery; however, re-sedation was observed. This finding persisted till 12 h after surgery, and the patient awakened on postoperative day 2.

**Conclusions:**

Remimazolam is a short-acting anesthetic, but long-term delayed emergence with re-sedation may occur even after flumazenil administration. Anesthesia using remimazolam requires anesthesia management that takes into account the individual differences in sensitivity and metabolism, with BIS as the indicator.

## Background

Remimazolam is an ultra-short-acting benzodiazepine anesthetic, and it is expected to be applied in management with the purpose of achieving early postoperative emergence as it can also be antagonized by flumazenil. However, 1.3–8.0% of delayed emergence has been reported in clinical trials in Japan [1, 2]. Herein, we report a case of long-term delayed emergence with re-sedation even after three times of flumazenil administration.

## Case presentation

We obtained the patient’s written consent for the publication of this case. The patient was a 71-year-old man with a height of 169.5 cm, body weight of 71.5 kg, and body mass index of 24.9 kg/m^2^. He was scheduled for a robotic-assisted laparoscopic radical prostatectomy following the diagnosis of prostate cancer. He had no history of medication prior to the surgery. His comorbidities were bilateral adrenal tumors (nonfunctional), bronchial asthma, and fatty liver. The blood test revealed decreased kidney function with a serum creatinine of 1.14 mg/dL and estimated glomerular filtration rate of 49.3 ml/min/1.73 m^2^, but no other abnormal findings including liver function.

**Fig. 1:**
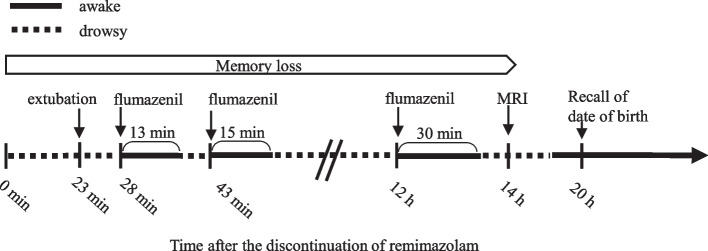
Changes in postoperative consciousness level over time. Arrows show flumazenil administration timings. Solid lines show awake periods, while dashed lines show drowsy periods

We initiated continuous administration of remimazolam 1 mg/kg/h and remifentanil 0.25 μg/kg/min after installing a bispectral index (BIS) monitor on the patient. We confirmed that the patient fell asleep during the administration of 4 mg remimazolam, after which the administration rate of remimazolam was reduced to 0.6 mg/kg/h and 50 mg rocuronium was administered. We subsequently adjusted the administration rates of remimazolam at 0.25–0.6 mg/kg/h and remifentanil aiming for BIS values of approximately 40–60. The patient’s mean blood pressure generally remained at ≥ 65 mmHg. We then administered remifentanil 0.3 μg/kg/min, and the BIS values were approximately 30–50. We gradually reduced remifentanil after the completion of the surgery, and the total amount of fentanyl administered during surgery was 300 μg. BIS value immediately before the completion of remimazolam administration was 61. Surgery time was 7 h and 35 min, and the total amount of remimazolam used was 346 mg. We confirmed the patient’s spontaneous breathing 1 min after the completion of the remimazolam administration. Even though the patient did not open his eyes to our call, his respiratory condition was stable; thus, we extubated him 23 min after the completion of the remimazolam administration. The patient’s breathing and circulation were stable even after extubation; however, he did not respond to a command. We administered 0.5-mg flumazenil 5 min after extubation. The patient promptly awakened and responded to a command after receiving 0.5 mg flumazenil, but subsequently became drowsy 13 min after flumazenil administration. We then administered 0.5 mg flumazenil, and the patient re-awakened. However, he became drowsy again 15 min after flumazenil administration. As the patient’s circulation and breathing were stable, he was placed under follow-up care at the ward. The patient was drowsy even in 12 h after surgery, and we administered flumazenil again. However, even though the patient’s consciousness level improved temporarily for 30 min, his re-sedation and amnestic symptoms persisted afterwards. Considering this, we suspected the presence of residual remimazolam. We performed a head MRI examination to search for the cause of delayed emergence, but no abnormal findings were observed. The patient could recall his birth date 17 h after surgery (Fig. 1). He could finally have normal conversations on postoperative day 2 and started taking meals and leaving his bed. The patient did not show any problems in particular problem; he was discharged on postoperative day 10 and he walked unaided.

## Discussion

Remimazolam is an ultra-short-acting benzodiazepine intravenous anesthetic, and early emergence and recovery from anesthesia are expected. However, a few percent of delayed emergence has been reported in clinical trials in Japan [1, 2], and coping with it is an important issue. This is possibly the first report on a case of delayed emergence up to a few days that cannot be fully antagonized by flumazenil after total intravenous anesthesia using remimazolam.

Remimazolam acts on the benzodiazepine binding site of γ-aminobutyric acid (GABA) A receptors to exhibit anesthetic and sedative effects. Remimazolam is rapidly metabolized into inactive metabolites by carboxylesterase mainly in liver tissues, and its duration of action is short. In addition, as the affinity of remimazolam metabolites to GABA receptors is extremely weak at approximately 1/400 of that of remimazolam, it is easy to predict recovery time [3, 4]. Furthermore, the half-life of remimazolam is hardly extended even in long-term administration [5]. However, 1.3–8.0% of delayed emergence has been reported in clinical trials in Japan [1, 2]. In cases with delayed emergence in the clinical trials, all patients awakened 1–2 min after the administration of flumazenil, and re-sedation was not reported. However, a case in which re-sedation was confirmed after anesthesia with remimazolam has also been reported [6], but the cause and frequency remain unknown.

In this case, the patient lost consciousness during the administration of 4 mg (0.055 mg/kg) remimazolam, and this dose was smaller less than the doses used in a Japanese phase IIb/III trial (0.17 ± 0.04 mg/kg) and a subsequent randomized controlled trial (0.16 ± 0.04 mg/kg) [1, 2]. Furthermore, even though the mean administration rate of remimazolam in anesthetic maintenance was 0.5 mg/kg/h, which was less than those of previous reports, BIS remained relatively low at 30–50. Based on these doses, we inferred that this patient had a high sensitivity to remimazolam. In fact, individual differences in sensitivity to benzodiazepine have been reported. In the report, 0.075 mg/kg midazolam was administered to healthy individuals, and the lowest modified Observer’s Assessment of Alertness/Sedation Scale score was within 3–4; however, individual differences in sedation scale were observed, and similar individual differences were also observed with remimazolam [3]. Chae reported the efficacy and safety of IV bolus remimazolam administration during anesthesia induction [7]. In this randomized controlled trial, both the 50% effective dose and 95% effective dose of remimazolam bolus dose causing loss of consciousness were lower in the population aged ≥ 65 years than in the population aged < 65 years. Moreover, they demonstrated a large margin of safety between loss of consciousness and hypotension. Thus, although there are individual differences in sensitivity to remimazolam, because there is little onset of hypotension with anesthesia using remimazolam, it was difficult to detect individual differences in remimazolam in hemodynamics.

One of the causes of delayed emergence is remimazolam overdose because the patient’s intraoperative BIS values remained lower than the range considered appropriate. During remimazolam administration, an electroencephalogram (EEG) showed an increased β-wave [8], and an increase in *β* wave is the calculation criteria for high BIS values. BIS values during remimazolam administration at appropriate doses were higher than those during other anesthetics, with some patients showing BIS > 60 [9]. Thus, the BIS values of 30–50 in this case may indicate an overdose of anesthesia using remimazolam. Furthermore, it has been reported that BIS values are weakly correlated with the depth of anesthesia when benzodiazepines are used instead of propofol [10], so it is preferable for anesthesiologists to interpret EEG waveforms without relying solely on BIS values, and a new EEG analysis algorithm during remimazolam use should be developed.

Another cause of delayed emergence is the effect of remimazolam on drug metabolism. Carboxylesterase, the metabolizing enzyme of remimazolam, is mainly found in the liver, and severe liver dysfunction causes delayed remimazolam metabolism [11]. Furthermore, carboxylesterase has interindividual variabilities and drug interactions [12]. Hydrolysis efficiency by carboxylesterase is higher in women than in men, and the difference was significant even when body weight differences had been corrected [13]. In vitro, diltiazem, one of the carboxylesterase inhibitors, exhibits an inhibitory effect on remimazolam metabolism; however, the concentration of diltiazem that inhibits remimazolam metabolism is above clinical dose; thus, it is considered clinically nonsignificant. In clinical situations, it is difficult to measure carboxylesterase activity because the collection of liver tissues is required for measuring carboxylesterase activity.

In this case, the patient awakened after flumazenil had been administered; however, he was resedated. One of the causes of re-sedation is attributable to the differences in the binding sites of benzodiazepine and flumazenil. Benzodiazepine anesthetics act on benzodiazepine binding sites that are situated at the extracellular domain (α-γ) interface and transmembrane domain (β-α, γ-β) interfaces, which are GABA_A_ receptor subunits. However, flumazenil acts on the extracellular domain (α-γ) interface of GABA_A_ receptors [14]. Flumazenil is believed to open the gap of the γ-β subunit interface, indirectly destabilizes the receptor structure and consequently antagonizes the action of benzodiazepine [15]. In other words, flumazenil does not directly antagonize benzodiazepine. However, under situations of long-term residual benzodiazepine, it can be a factor of delayed re-sedation and amnestic symptoms, as observed in the present case.

In the present report, we performed total intravenous anesthesia using remimazolam in a robotic-assisted laparoscopic radical prostatectomy and encountered a case of long-term delayed emergence that cannot be fully antagonized by flumazenil. The cause of postoperative delayed emergence could be individual differences in sensitivity, and the cause of re-sedation could be the action mechanism of flumazenil, but case accumulation and further studies are warranted.

## Data Availability

The data used in this case report are available from the corresponding author on reasonable request.

## Abbreviations

BIS: Bispectral index
EEG: Electroencephalogram
GABA: γ-Aminobutyric acid
